# Pyoderma gangrenosum triggered by secukinumab in a patient with palmoplantar pustulosis^☆^

**DOI:** 10.1016/j.abd.2023.06.012

**Published:** 2024-09-02

**Authors:** Huizhong Wang, Jingru Sun

**Affiliations:** a Department of Dermatology and Venereology, Peking University First Hospital, Beijing, China; b Beijing Key Laboratory of Molecular Diagnosis on Dermatoses, Beijing, China; c National Clinical Research Center for Skin and Immune Diseases, Beijing, China; d NMPA Key Laboratory for Quality Control and Evaluation of Cosmetics, Beijing, China

Dear Editor,

A 35-year-old female patient presented with recurrent multiple pustules involving her palms and soles for more than one year (Fig. 1A). No acne, keratotic plaques, or bone-joint manifestations were reported. A skin biopsy taken from her palm showed a collection of neutrophils within the spongiform epidermis (Fig. 1B‒C). Thus, a diagnosis of palmoplantar pustulosis (PPP) was made. As she could not tolerate the side effects of conventional systemic drugs, secukinumab was chosen as an alternative therapy. Her pustular lesions on the palms and soles almost healed after 3 months of treatment (secukinumab 300 mg per week for 5 weeks, followed by secukinumab 300 mg per month, Fig. 1D). However, several painful eruptions started on her lower legs after 6 doses of secukinumab. The lesions rapidly ulcerated and enlarged after the 7th dose of secukinumab. Fever or other systemic symptoms were absent. She had a history of rheumatic heart disease with normal cardiac function for 10 years, controlled by 4 mg methylprednisolone every day. No history of inflammatory bowel disease was reported.

**Figure 1:**
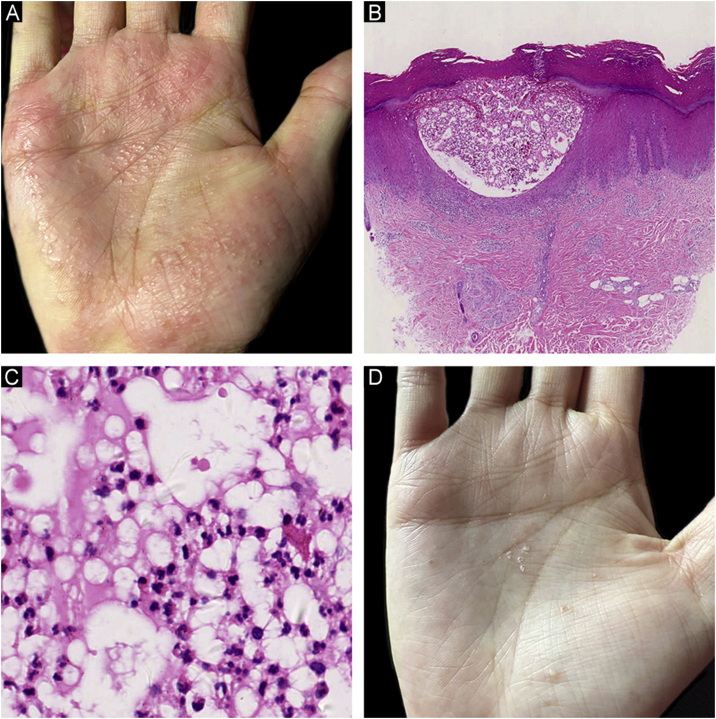
Clinical and histological findings of palmoplantar pustulosis (PPP). (A) Multiple pustules and pustule-vesicles with ill-circumscribed erythemas on the right palm before secukinumab treatment; (B) A collection of neutrophils within spongiform epidermis in a lower power view of PPP (Hematoxylin & eosin, ×20); (C) A collection of neutrophils in a higher magnification of PPP (Hematoxylin & eosin, ×200); (D) Scattered pustules with focal desquamation after secukinumab treatment.

On physical examination, multiple tender wide ulcers with irregular elevated violaceus borders surrounded by infiltrated erythema were found on her lower extremities (Fig. 2A). The largest ulcer was approximately 6 cm in diameter (Fig. 2B). Repeated swab cultures for bacteria, fungi, and mycobacteria from these ulcers were all negative. Hematoxylin-eosin staining of skin biopsy revealed a predominantly neutrophilic infiltrate in the dermis (Fig. 3A‒B). In addition, laboratory examinations revealed normal complete blood count and moderately elevated levels of erythrocyte sedimentation rate (56 mm/h, normal range 0‒20 mm/h), C-reactive protein (37.6 mg/L, normal range <10 mg/L), and anti-streptolysin O (438 IU/mL, normal range <200 IU/mL). A blood test for HLA-B51 was positive. Based on the clinicopathological findings, this patient was diagnosed with secukinumab-induced pyoderma gangrenosum (PG). Then, we discontinued her anti-IL17A treatment and started oral methylprednisolone (24 mg/day), oral sulfasalazine (3 g/day), and a careful ulcer care. Lesions ulcers improved substantially after 8 weeks (Fig. 4A‒B).

**Figure 2:**
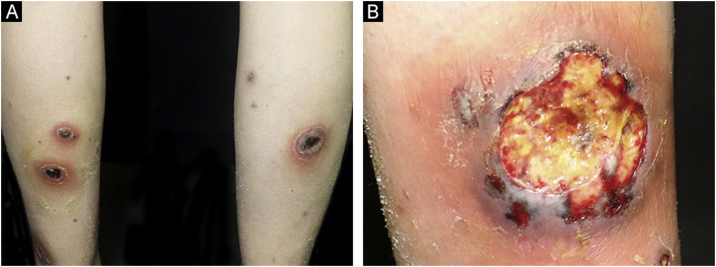
(A) Multiple ulcers with erythematous raised borders on the posterior aspects of both lower extremities. (B) The largest ulcer had a diameter of 6 cm adjacent to the right ankle.

**Figure 3:**
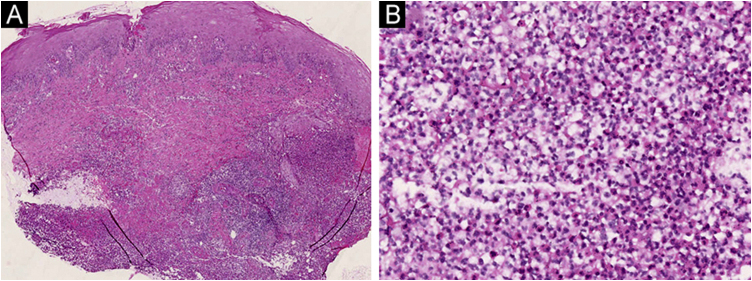
(A) A skin biopsy taken from the edge of the ulcer demonstrated diffuse inflammatory cells infiltrated throughout the whole dermis. (Hematoxylin & eosin, ×20); (B) Neutrophils represented the predominant infiltrating cells. (Hematoxylin & eosin, ×200).

**Figure 4:**
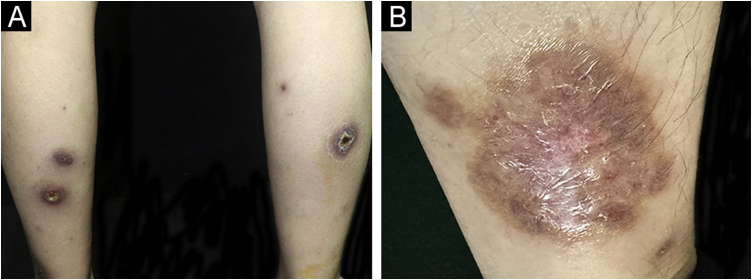
(A-B) The ulcers almost resolved after eight weeks of treatment.

PPP is characterized by chronic recurrent sterile pustules on the palms and soles. Several studies have proposed that IL-17 may be a dominant cytokine in the pathogenesis of PPP.^1^, ^2^ Thus, anti-IL-17 biologics may represent an effective therapeutic option. As expected, our patient’s pustular lesions resolved rapidly in response to secukinumab therapy. However, PG, an uncommon ulcerating inflammatory skin disease, also developed rapidly after 7 doses of secukinumab.

PG is a rare neutrophilic dermatosis, featured by progressive painful ulcers.^3^ Although a variety of cytokines, including TNFα, IL-8, IL-17, and some chemokines were elevated in PG, the exact pathogenesis of PG remains unclear.^3^ Previous literature documented some recalcitrant PG cases were successfully treated with secukinumab.^4^, ^5^ However, there are also several reported cases of PG being paradoxically induced by IL-17 inhibitors.^6^, ^7^, ^8^ Unlike previous reports, our case represented an exceptional secukinumab-induced PG occurring in a PPP patient with HLA-B51 positivity. It is assumed that IL-17 inhibitors showed a “potential double pathogenetic face” in some inflammatory skin diseases. They have both an ameliorating effect and a paradoxical exacerbating effect. A compensatory increase in IL-23 or interferon-α induced by IL-17 inhibition might play a significant role in the pathogenesis of PG.^4^, ^8^ Furthermore, our patient’s HLA-B51 genetic background might have contributed to the development of PG, because HLA-B51 is associated with neutrophil hyperfunction.^9^ Thirdly, PPP has been suggested as a distinct entity from pustular psoriasis or palmoplantar psoriasis with a poorly understood pathophysiology.^10^ Whether the patients diagnosed with PPP can respond well to IL-17 inhibitors still needs validation by large cohorts.

Overall, the marked improvement observed in our patient with PPP suggests that IL-17 inhibitor may be a promising therapeutic option for PPP. However, clinicians should also be aware of the potentially paradoxical PG-like drug eruption.

## Financial support

This study was supported by the 10.13039/501100001809National Natural Science Foundation of China (82002903, J.S.).

## Authors’ contributions

Huizhong Wang: The study concept and design; literature search; data collection; critical review of the literature; writing of the manuscript or critical review of important intellectual; final approval of the final version of the manuscript.

Jingru Sun: The study concept and design; critical review of the literature; writing of the manuscript or critical review of important intellectual; final approval of the final version of the manuscript.

## Conflicts of interest

None declared.
